# The continuous performance test (rCPT) for mice: a novel operant touchscreen test of attentional function

**DOI:** 10.1007/s00213-015-4081-0

**Published:** 2015-09-29

**Authors:** Chi Hun Kim, Martha Hvoslef-Eide, Simon R. O. Nilsson, Mark R. Johnson, Bronwen R. Herbert, Trevor W. Robbins, Lisa M. Saksida, Timothy J. Bussey, Adam C. Mar

**Affiliations:** 1 Department of Psychology and MRC/Wellcome Trust Behavioural and Clinical Neuroscience Institute, University of Cambridge, Downing St, Cambridge, CB2 3EB UK; 2 Academic Obstetrics and Gynaecology, Department of Surgery and Cancer, Chelsea and Westminster Hospital, Imperial College London, SW10 9NH London, UK; 3 Department of Neuroscience and Physiology Neuroscience Institute, New York University, New York, NY USA

**Keywords:** Mouse, Continuous performance test, Touchscreen operant chamber, Mouse strain differences, Donepezil

## Abstract

**Rationale:**

Continuous performance tests (CPTs) are widely used to assess attentional processes in a variety of disorders including Alzheimer’s disease and schizophrenia. Common human CPTs require discrimination of sequentially presented, visually patterned ‘target’ and ‘non-target’ stimuli at a single location.

**Objectives:**

The aims of this study were to evaluate the performance of three popular mouse strains on a novel rodent touchscreen test (rCPT) designed to be analogous to common human CPT variants and to investigate the effects of donepezil, a cholinesterase inhibitor and putative cognitive enhancer.

**Methods:**

C57BL/6J, DBA/2J and CD1 mice (*n* = 15–16/strain) were trained to baseline performance using four rCPT training stages. Then, probe tests assessed the effects of parameter changes on task performance: stimulus size, duration, contrast, probability, inter-trial interval or inclusion of flanker distractors. rCPT performance was also evaluated following acute administration of donepezil (0–3 mg/kg, i.p.).

**Results:**

C57BL/6J and DBA/2J mice showed similar acquisition rates and final baseline performance following rCPT training. On probe tests, rCPT performance of both strains was sensitive to alteration of visual and/or attentional demands (stimulus size, duration, contrast, rate, flanker distraction). Relative to C57BL/6J, DBA/2J mice exhibited (1) decreasing sensitivity (*d*′) across the 45-min session, (2) reduced performance on probes where the appearance of stimuli or adjacent areas were changed (size, contrast, flanking distractors) and (3) larger dose- and stimulus duration-dependent changes in performance following donepezil administration. In contrast, CD1 mice failed to acquire rCPT (stage 3) and pairwise visual discrimination tasks.

**Conclusions:**

rCPT is a potentially useful translational tool for assessing attention in mice and for detecting the effects of nootropic drugs.

## Introduction

The continuous performance test (CPT) has been widely used to assess deficits in attentional function in many neurodegenerative and neuropsychiatric disorders including Alzheimer’s disease (Perry and Hodges 1999; Stopford et al. 2012) and schizophrenia (Cornblatt et al. 1989; Cornblatt and Malhotra 2001). In the original version (X-CPT), Rosvold et al. (1956) asked subjects to monitor a sequentially presented series of letters on a monitor and to respond only when the letter (‘X’) appeared. Numerous versions of CPT have been introduced that vary cognitive load such as working memory and/or perceptual load (e.g. AX-CPT: Rosvold et al. (1956), ‘identical pairs’ CPT: Cornblatt et al. (1989), ‘noisy stimulus’ CPT: Nuechterlein et al. (1983)). However, a common feature of most CPT variants is that subjects are asked to report or respond to designated ‘target’ stimuli and to ignore or inhibit responding to designated ‘non-target’ stimuli.

To successfully translate results from preclinical work to the clinic, comparable and sensitive tests in animals are essential. To this end, devising attentional tasks with analogous features in both humans and animals can help maximise the chances of successful translation across species in preclinical and clinical stages of drug development (Nuechterlein et al. 2009; Lustig et al. 2013). Mouse versions of cognitive tests are particularly important, as many disease models are currently available only in mice (Papaleo et al. 2012; Webster et al. 2014).

Several tests have been adapted or developed to assess attentional function in mice such as the 5-choice serial reaction time task (5-CSRTT) (Humby et al. 1999; Romberg et al. 2011), the 5-choice continuous performance task (5C-CPT) (Young et al. 2009) and the sustained attention task (SAT) (St. Peters et al. 2011). The 5-CSRTT and the SAT were originally developed for rats and are well-validated based on a wealth of data examining its behavioural, neural and pharmacological underpinnings (Carli et al. 1983; McGaughy et al. 1996; Lustig et al. 2013). Recent attempts have also been made to more specifically translate the 5-CSRTT (Voon et al. 2014), 5C-CPT (van Enkhuizen et al. 2014) and SAT (Demeter et al. 2008) tasks to humans. Akin to human CPTs, these tests share the common elements that animals are required to pay attention across a series of trials and to detect and accurately respond to the presence of a designated target stimulus. Moreover, individual tasks have other features in common with human CPT; for example, ‘non-target’ trials requiring an alternative response are incorporated in the 5C-CPT and SAT paradigms.

Despite the similarities of these tasks to commonly used variants of the human CPT paradigm, there are also important differences. For instance, in both the 5-CSRTT and 5C-CPT, animals scan a horizontal array of possible locations where the target might be presented, which requires spatially divided attention. More critically, the 5-CSRTT, 5C-CPT and SAT all only require that the subject report the detection (e.g. presence or absence) or spatial location (e.g. which aperture is lit) of a simple light stimulus. Common versions of the CPT, however, typically require discrimination of target stimuli from a set of non-target stimuli that share a number of overlapping features (e.g. colour, brightness, contrast). In other words, over and above a simple detection of a light, subjects are required to discriminate, identify and/or recognize a visual target pattern with reference to a learned pattern. The added difficulty of having to discriminate and remember target patterns has been demonstrated to be a key variable for observing vigilance decrements in perceptual sensitivity within the human CPT paradigm (Parasuraman 1979). Moreover, the capacities for simple light detection or spatial localisation likely require and/or recruit distinct neural pathways and cognitive/perceptual processes from those tapped by more complex visual discrimination/identification/recognition (Lashley 1931; Petruno et al. 2013; Schneider 1969). The abilities to separate and remember visual input patterns are thus potentially important cognitive processes to consider in the translation of rodent attentional tasks to many common human CPT paradigms.

In this study, we implemented a recently developed rodent touchscreen test (rCPT, Mar et al., unpublished), designed to be analogous to the common human CPT versions, to investigate the attentional performance of several mouse background strains. Similar to many human CPTs, optimal rCPT performance requires mice to respond to ‘target’ visual pattern stimuli and to withhold responses to ‘non-target’ stimuli during sessions in which both stimulus types are presented sequentially at a single screen location. C57BL/6J (C57), DBA/2J (DBA) and CD1 mouse strains were selected for study based on the fact that they are some of the most commonly used background strains in neuroscience research (e.g. knock-out, transgenic, optogenetic or chemogenetic manipulations). Additionally, the attentional performance of one or all of the strains has been previously examined and/or compared within mouse versions of the 5-CSRTT, 5C-CPT or SAT (Greco et al. 2005; Patel et al. 2006; Young et al. 2009; Oliver et al. 2009; St. Peters et al. 2011), providing points of reference for comparison. To help behaviourally validate the rCPT, performance was examined following changes to various task parameters (e.g. stimulus size, duration, contrast, target stimulus probability and inter-trial interval), or under challenging conditions (e.g. flanking distractors)—manipulations that are often implemented in human studies. Finally, as visual and attentional performance is known to be affected by cholinergic manipulations (McGaughy et al. 1996, 2002; Dalley et al. 2004; Howe et al. 2013; Pinto et al. 2013; Berry et al. 2014), rCPT performance was evaluated following the acute, systemic administration of donepezil. Donepezil is a cholinesterase inhibitor that has been shown to improve attentional performance in humans (Sahakian et al. 1993; Singh et al. 1996; Rockwood et al. 2004; Foldi et al. 2005; Bentley et al. 2008) and in rodents (Muir et al. 1994; Balducci et al. 2003; Romberg et al. 2011), particularly in subjects having low baseline cognitive performance and in tasks which require an elevated cognitive/attentional load (Van Dam et al. 2005; Robbins 2000).

## Methods and materials

### Subjects

Male C57BL/6J (C57, *n* = 16, Charles River, UK), DBA/2J (DBA, *n* = 16, Harlan, UK) and CD1 (*n* = 15, Charles River, UK) mice were 7–9 weeks old at the start of behavioural procedures. Mice were housed between one and four per cage, in a holding room with a 12-h light cycle (lights off at 7 AM). All experiments were performed during the dark cycle. After a minimum of 7 days of acclimatisation to the animal facility, food was restricted to maintain 85–90 % of free feeding body weight with ad libitum drinking water throughout the experiments. During food restriction, reward pellets (14 mg Bio-Serv purified rodent dustless precision pellets through Sandown Scientific) were introduced in the home cage for minimum 3 days to decrease neophobia to the rewards during training. All experiments were in compliance with the United Kingdom Animals (Scientific Procedures) Act (1986).

### Apparatus

**Fig. 1:**
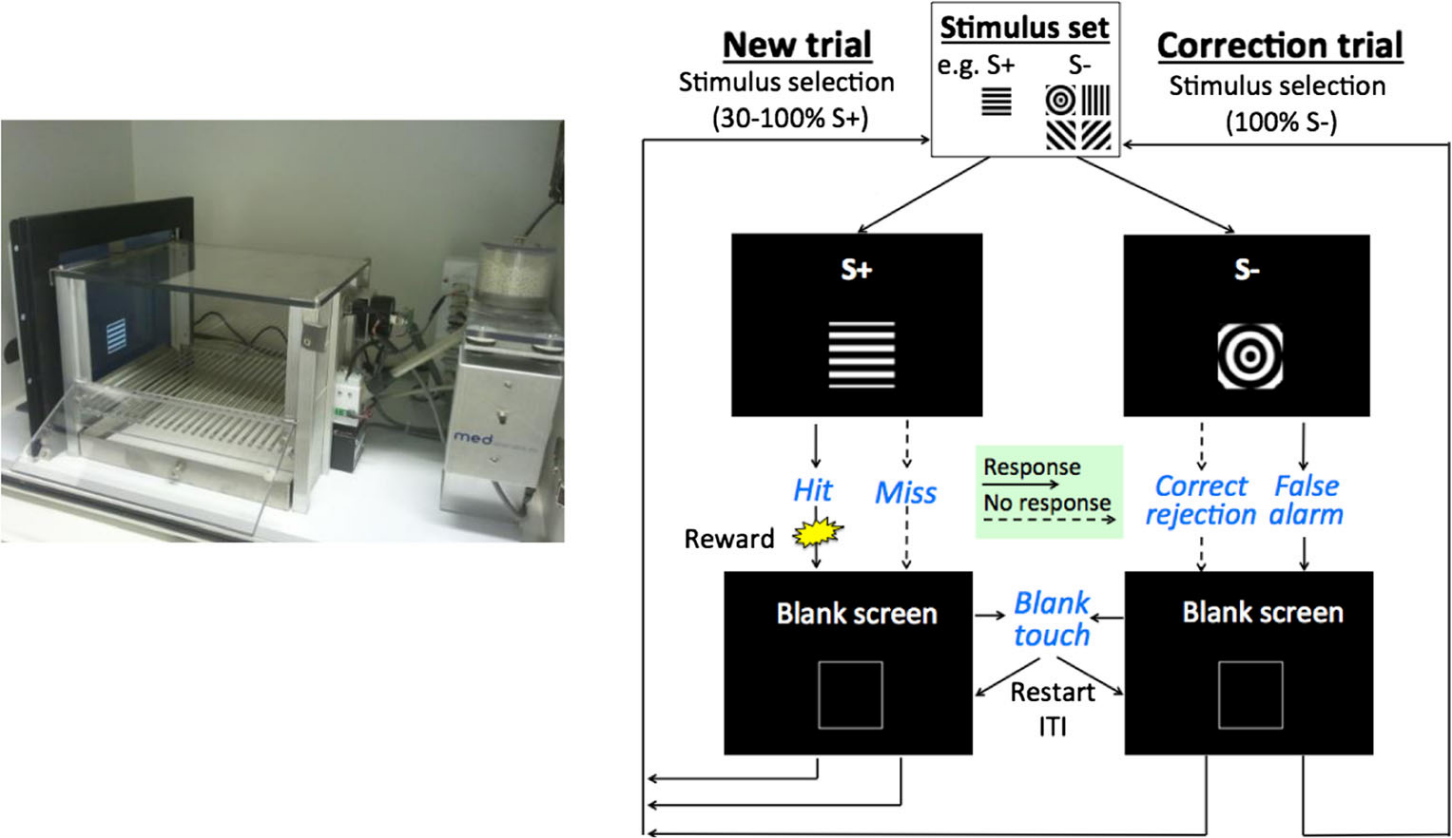
Photograph of the touchscreen operant chamber and a flowchart of the rodent continuous performance test (rCPT: modified from Mar et al., unpublished). *Left*: The chamber was equipped with a touchscreen on one end and a reward delivery magazine/tone generator on the other. *Right*: On each trial, either a target (S+) or non-target (S−) stimulus was presented within a *white frame* (3.5 × 3.5 cm) in the horizontal centre of the screen, 2 cm above the grid floor: After touching the stimulus or the stimulus duration period ends, a blank screen with a central *white frame* was presented during an inter-trial interval (ITI) preceding the next trial. *Blue italics* indicate a touch response made by the mouse, while *black non-italics* indicate events triggered by the computer program. *Solid* and *dotted short arrows* indicate a mouse touch response or no response to the stimulus, respectively. A ‘hit’ is a response to the S+, a ‘miss’ is no response to the S+, a ‘correct rejection’ is no response to the S and a ‘false alarm’ is a response to the S−. A hit resulted in reward delivery, with reward collection triggering a brief (5 s) ingestion delay period prior to a new trial ITI. A miss or a correct rejection was followed by a new trial ITI, while a false alarm was followed by a correction trial ITI. Touches within the *white frame* during the ITI reset the ITI, effectively delaying the onset of the next stimulus

Behavioural tests were conducted in touchscreen operant chambers (see Fig. 1 left; modified from Med Associates Inc., St. Albans, VT) which have been described in other reports (Romberg et al. 2011; Mar et al. 2013). In brief, the operant chamber had a rectangular shape with a touchscreen on one end and a reward delivery magazine fitted to a metal wall on the opposite end. The chamber also contained a house light, a tone generator, a metal grid floor and clear Perspex walls. A box with a ventilating fan housed the chamber to attenuate outside sound and light. Custom software written (by ACM) in Visual Basic 2010 Express .NET (Microsoft, 2010) was used to run the behavioural program controlling the apparatus and to record the data.

### Behavioural procedures

#### Rodent continuous performance test—task training

Behavioural testing began with two sessions of 20-min habituation to the chamber. During these sessions, the chamber was left dark with 10 reward pellets in the reward magazine. Following habituation, mice underwent four stages of rCPT training (see Table 1).

**Table 1:**
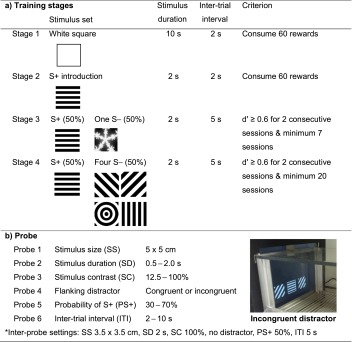
rCPT training stages and probe tests. (a) rCPT training consisted of four stages and mice progressed between stages by reaching stage-specific criteria. S+ denotes the target stimulus and S− denotes the non-target stimulus. ‘50 %’ in stages 3 and 4 refers to the probability of presentation of either an S+ or an S− trial within a session. (b) Six probe tests were implemented sequentially after completion of stage 4 training. Inter-probe sessions, using stage 4 parameter settings, were run between distinct probe sessions. The photo depicts an example of an incongruent flanking distractor trial: a central stimulus (horizontal lines) in the centre and two incongruent distractors (diagonal lines), one on either side

In stage 1, mice were trained to touch a white square presented within a white-outlined frame (3.5 × 3.5 cm) centred horizontally on the screen, with the bottom of the stimulus positioned 2 cm above the grid floor. A limited hold (LH) period was defined as the duration of stimulus presentation (SD)—at this stage 10 s—plus an additional 0.5-s interval after stimulus removal to help account for responses initiated prior to offset. If a touch response was made within the white frame during the LH period, the white square stimulus was removed from the display (if present) and reward pellet delivery was paired with a 1-s tone and illumination of the magazine light. A head entry into the magazine to collect the reward turned off the magazine light and initiated an inter-trial interval (ITI) of 2 s. Following the ITI, the next trial began with the presentation of the white square. If no touch response within the frame occurred during the LH period, the ITI prior to the next stimulus was initiated. A touch within the white outline frame during the ITI period (‘blank touches’) reset the ITI, i.e. delaying the start of the next stimulus presentation. The criterion set for stage 1 was for mice to earn 60 rewards within the 45-min session. The session was terminated either when 45 min had elapsed or when the maximum rewards of 100 were collected. From stages 1 to 4, when a mouse reached the performance criterion for a given stage, it was advanced to the next stage.

In stage 2, the white square stimulus was replaced by an S+ stimulus and the SD reduced to 2 s (LH = 2.5 s). The S+ stimulus was either a horizontal or vertical line stimulus and was counterbalanced across individuals within each strain. From stage 2 onwards, a head entry into the magazine for reward collection initiated a brief ingestion delay (ID) period of 5 s before proceeding to the next ITI. All other parameters and performance criteria were identical to those of stage 1.

In stage 3, a non-target S**−** stimulus was added to the stimulus set (the counterbalanced target S+ from stage 2 and one novel ‘snowflake’ S**−**) and randomly presented on 50 % of trials. In this and all subsequent stages, stimuli were luminance-matched in attempt to equate stimulus salience. The ITI was also increased to 5 s. A touch to an S**−** stimulus during the LH period resulted in stimulus removal (if present) followed by an ITI period prior to a correction trial. On correction trials, the presented stimulus was always the S**−** and consecutive correction trials continued until no response to an S**−** was made during the LH period. Correction trials, together with ‘blank touches’ resetting the ITI (see stage 1), were included to discourage non-selective responding to the stimulus and screen, respectively. All other settings were identical to those of stage 2. The criterion for this two-choice visual discrimination phase was a performance sensitivity (*d*′—see data analysis section) of above 0.6 for two consecutive sessions. All animals were run a minimum of seven sessions in stage 3 to establish an acquisition curve. Upon discovering that the CD1 group was not able to meet the criteria for stage 3, alternate parameters (SD 3 and ITI 4 s) for CD1 were conducted in an attempt to improve performance levels.

In stage 4, the ‘snowflake’ S**−** was no longer used and replaced by four new S**−** stimuli, thus forming a stimulus set of one S+ and four S**−** stimuli. As with stage 3, the probability of presentation of an S+ or an S**−** on any given trial was 50 % each. All other settings were the same as stage 3, with S**−** choices resulting in correction trials using a stimulus randomly selected from the 4 S**−** stimuli. The performance criterion for this stage was maintained at a sensitivity (*d*′—see data analysis section) of above 0.6 for two consecutive sessions. All animals were run a minimum of 20 sessions in stage 4 to establish an acquisition curve to ‘baseline’ performance.

#### Rodent continuous performance test—probe tests

Six different types of probe tests were used to investigate how alterations of certain task parameters affected rCPT performance (see Table 1). Probe sessions were implemented after all mice had reached stage 4 criterion, and group performance of each strain was stable for three consecutive sessions, i.e. no significant main effect of or interaction for block for *d*′ across two consecutive 3-session blocks. Before commencing probe sessions, the ID period was increased from 5 to 8 s as some mice were observed to spend longer than 5 s to consume a reward. Between each probe, baseline rCPT performance was re-established by running a minimum of two inter-probe baseline sessions using the stage 4 parameter settings.

Probe 1 (Stimulus Size) investigated whether a larger stimulus size would improve performance (*d*′) in two strains. The stimulus size was increased from 3.5 × 3.5 to 5 × 5 cm and subsequently reduced back to the original size to help rule out any effects due to prolonged training per se. To increase size, the same stimuli were expanded to fill a larger central square. Thus, in addition to making the stimuli more salient (more white pixels over a greater area), it also effectively lowered the spatial frequency of the stimuli, thus changing its appearance. The final three sessions (e.g. stable performance) in each stimulus size condition was averaged for analysis.

In probe 2 (Stimulus Duration), the SD was reduced to increase the attentional load of the task—reducing the stimulus presentation time is a classic manipulation to tax attention in human studies. Two types of variable SD sessions were run, for three sessions each, in a random order. One variable SD session type was composed of 1-, 1.5- and 2-s SDs and another composed of 0.5-, 0.75- and 1-s SDs. For all of the variable SD sessions, the LH remained constant at 2.5 s giving the animals an identical amount of time to execute their responses and maintaining a similar event rate. Performance across three sessions for each SD was averaged for analysis. As the 1-s SD was presented in both variable SD sessions, it was averaged across six rather than only three sessions.

In probe 3 (Stimulus Contrast), stimulus contrast was altered by adjusting the difference in luminance between adjacent dark and light stripes in each of the stimuli, keeping the overall luminance of the stimulus the same (e.g. 100 % contrast = black versus white, 0 % contrast = entire stimulus 50 % grey). This probe thus assessed task performance under more demanding perceptual conditions, with the additional requirement of stimulus generalization (i.e. similar response despite different visual appearance of stimuli). Two types of variable stimulus contrast (SC) session types were used in a similar manner as probe 2. One session type had 50, 75, 87 and 100 % SC and the other had 12.5, 25, 50 and 100 % SC. Performance across three sessions at each SC was averaged for analysis, except for 100 % SC which was averaged across six sessions.

In probe 4 (Flanker Distractors), we investigated whether simultaneously presented flanking distractors would decrease attentional performance. Successful attentional function requires inhibition of potentially distracting information. This probe was devised as a variant of the Eriksen Flanker task (Eriksen and Eriksen 1974) and assessed the impact of adjacent response-compatible or response-incompatible visual distractors on performance of the rCPT. The flanking distractor session was comprised of three trial types: no distractor, congruent and incongruent distractor. No-distractor trials were identical to stage 4 baseline parameters, i.e. inter-probe baseline. Congruent distractor trials had two identical stimuli (3.5 × 3.5 cm) presented as flankers to the central stimulus, that were of the same type as the central stimulus (e.g. all S+ or all S**−**). These response-compatible, i.e. congruent, flankers are often found to enhance task performance (*d*′, response latency). Incongruent distractor trials used the same task parameters as congruent distractor trials, except that the central stimulus was of a different type relative to the two identical flankers (e.g. central S+ with two identical S**−** flankers or central S**−** with two identical S+ flankers). These response-incompatible, i.e. incongruent, flankers are often found to reduce task performance (*d*′, response latency).

In probe 5 (Stimulus Probability), the probability of presentation of the S+ stimulus (PS+) trials (relative to S**−** stimulus trials) was varied. After two sessions with the baseline PS+ of 50 %, two sessions for each of the PS+ 70 and PS+ 30 % conditions were given with two sessions of inter-probe baseline between. The average of the two sessions for each PS+ was used for analysis, except for PS+ 50 % which included four sessions.

In probe 6 (Event Rate), the ITI was manipulated to change the interval between two stimulus presentations. Low and high event rates have both been shown to reduce sustained attentional performance (e.g. decrements across a session) within the human CPT paradigm. Two ITI conditions, 2 and 10 s, were tested in the same manner with probe 5. The probe began with two sessions of baseline 5-s ITI which were followed by two sessions of 2-s ITI. Then following two sessions of inter-probe baseline 5-s ITI, two sessions of 10-s ITI were run. The average of the two sessions was used for analysis, except for the 5-s ITI which included four sessions.

#### Pairwise visual detection and discrimination tasks

The rCPT task was designed to test attention with brief presentations of individual visual stimuli on the screen over a lengthy, sequential series of trials. Subjects are required to initiate a touch response upon seeing an S+ while withholding such responses in the presence of an S**−** (e.g. rapid yes/no visual detection and discrimination task). By contrast, pairwise visual detection and discrimination tasks assess these visual capacities using a comparison design (e.g. simultaneous presentation of S+ and S**−** stimuli on each trial). In these tasks, subjects are required, on each trial, to orient and touch the screen location (left or right) where the S+ is presented while not responding to the side containing the S**−** (e.g. two-alternative forced choice tasks). The attentional load is thus reduced in the standard version of the pairwise visual detection and discrimination task, with no time limitations imposed on stimulus presentation or responding on each trial and the total number of trials limited to 30 (non-correction) per session. Moreover, memory load is reduced in the pairwise discrimination task relative to rCPT as both S+ and S**−** options are presented on each trial.

For the C57 and DBA strains, visual detection training began after finishing rCPT baseline training and all probe test sessions. We modified the previously described pretraining and pairwise visual discrimination training procedures (Horner et al. 2013) for the current experiment. Briefly, in the pairwise visual detection and localisation task a white square was presented in one of two response windows (each 3.5 × 3.5 cm) and remained on the screen until a mouse responded to one of the windows. When a mouse touched the stimulus, the stimulus was removed and a reward pellet was delivered with a tone and illumination of the magazine light. A magazine head entry for reward collection turned off the magazine light and initiated a 5-s ITI. A response to the blank response window removed the stimulus and resulted in a 5-s time-out period with illumination of the house light, followed by 1-s ITI. Following an incorrect response, a correction trial procedure was used in which the stimulus appeared in the same response window as the previous trial. These correction trials were repeated until a mouse made a response to the stimulus and were not counted towards the total trial number. The session finished after either completion of 30 trials or 60 min, whichever occurred first.

The pairwise visual discrimination task began when all mice reached the criterion of over 80 % correct for two consecutive sessions in the visual detection task. Two stimuli from rCPT stage 4—one S+ and one S**−** (horizontal and vertical lines) with the same stimulus size and luminance—were used to keep the visual demands as similar as possible. The two stimuli were always presented simultaneously within the two response windows, and the side of the correct stimulus was pseudorandomly selected on each trial such that they did not appear on the same side for more than three consecutive trials. Other parameters were the same as in the visual detection task.

For CD1 mice, most procedures were the same as those used for the other two strains. The primary difference was that the two stimuli used for the pairwise visual discrimination task were from rCPT stage 3 instead of stage 4, as these mice did not experience stage 4. Also the size of the stimuli was increased to 7 × 7 cm in the later stage of pairwise visual discrimination training in attempt to improve task performance.

#### Drug preparation and injections

Donepezil hydrochloride (Toronto Research Chemicals, Toronto) was dissolved in physiological saline each day for intraperitoneal (i.p.) injections. Mice were given injections (0.1 ml/10 g body weight) an hour before the rCPT testing session (mixed 1- and 4-s SD). Two dosing schedules were used which were balanced for strain and dose per each day: one with saline, 0.03-, 0.1- or 0.3-mg/kg donepezil, the other with saline or 3-mg/kg donepezil. The doses of donepezil were chosen based on a previous mouse touchscreen 5-CSRTT study (Romberg et al. 2011). In addition, as cholinergic drugs have been previously demonstrated to exert differential effects based on task difficulty, we used both easier (4-s SD) and harder (1-s SD) conditions within a session instead of 2-s SD used during the baseline training. Between injections, there was a minimum of two drug-free sessions on the stage 4 ‘baseline’ parameter settings.

### Data analysis

#### Measurements for rCPT

Four basic measures were assessed based on animals’ responses and stimulus types: response to the target stimulus S+ (hit), no response to the S+ (miss), response to a non-target stimulus S**−** (false alarm: FA) or no response to an S**−** (correct rejection: CR). These parameters were combined to calculate hit and false alarm rates:

$$ \begin{array}{l}\mathrm{Hit}\kern.2em \mathrm{rate}\kern.2em \left(\mathrm{H}\mathrm{R}\right)=\frac{\mathrm{Hit}}{\mathrm{Hit}+\mathrm{Miss}}\kern1em \\ {}\mathrm{False}\kern.2em \mathrm{alarm}\kern.2em \mathrm{rate}\kern.2em \left(\mathrm{FAR}\right)=\frac{\mathrm{False}\kern.2em \mathrm{alarm}}{\mathrm{False}\kern.2em \mathrm{alarm}+\mathrm{Correct}\kern.2em \mathrm{rejection}}\kern1em \end{array} $$

Relying on these two parameters in isolation may lead to misinterpretation of task performance. For example, a high HR does not always indicate a good score—if combined with an equally high FAR, it is likely indicative of high levels of non-selective responding rather than selective responding to the target stimulus. For this reason, composite measures of HR and FAR were used based on detection theory (Green and Swets 1966; Frey and Colliver 1973; Macmillan and Creelman 2004): two sensitivity (*d*′ and SI) and two response bias (*c* and RI) indices. Sensitivity refers to the perceptual discriminability between the S+ and S**−**; i.e. higher values indicate better visual discrimination. Response bias refers to the criterion or willingness to make responses, e.g. conservative (high *c* or low RI values) or liberal (low *c* or high RI values) strategies. *d*′ and *c* are considered as parametric indices that are used under assumptions such as normal distributions and equal variances for the discriminability of targets and non-targets. SI and RI are generally described as non-parametric indices which do not rely as heavily on these assumptions (Frey and Colliver 1973; Stanislaw and Todorov 1999).

$$ \begin{array}{ll}{d}^{\prime }=z\left(\mathrm{H}\mathrm{R}\right)-z\left(\mathrm{FAR}\right)\hfill & \mathrm{Sensitivity}\ \mathrm{index}\left(\mathrm{S}\mathrm{I}\right)=\frac{\mathrm{HR}-\mathrm{FAR}}{2\left(\mathrm{H}\mathrm{R}+\mathrm{FAR}\right)-{\left(\mathrm{H}\mathrm{R}+\mathrm{FAR}\right)}^2}\hfill \\ {}c=-\frac{z\left(\mathrm{H}\mathrm{R}\right)-z\left(\mathrm{FAR}\right)}{2}\hfill & \mathrm{Responsivity}\kern0.5em \mathrm{index}\left(\mathrm{R}\mathrm{I}\right)=\frac{\mathrm{HR}-\mathrm{FAR}-1}{1-{\left(\mathrm{H}\mathrm{R}-\mathrm{FAR}\right)}^2}\hfill \end{array} $$

Additionally, the following measures were recorded: ‘blank’ touches to the empty frame during the ITI and latencies for reward collection, correct and incorrect responses. To assess sustained attention across time bins, a 45-min session was divided into 3 × 15-min time bins.

#### Statistical analysis

All data were checked for normal distribution by the Shapiro-Wilk test and for homogeneity of variances by Levene’s test. Based on the results, independent samples *t* tests, Mann-Whitney *U* tests or Kruskal-Wallis tests were used, as appropriate. A one-sample, one-tailed *t* test was used to compare CD1 stage 3 rCPT performance relative to chance levels. Within-subject factors were subjected to repeated measures ANOVA (rmANOVA). Any violations of sphericity were corrected using the Greenhouse-Geisser (GG) method. For main effects, post hoc analysis was performed with the Bonferroni correction. Three-way interactions were decomposed by simple two-way interaction analyses at each level of the variables. When two-way interactions were found, simple main effects analysis for either within- or between- subject factors was performed using a Sidak correction. All statistical analyses were conducted with SPSS version 22.

## Results

### rCPT training stages and baseline performance

#### rCPT training stages 1 and 2: simple detection of white square and S+

**Fig. 2:**
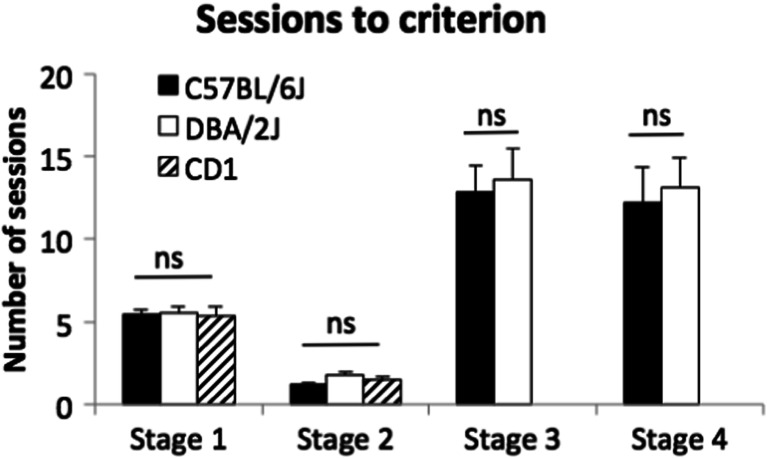
Sessions to criterion for each rCPT training stage. The parameters and criteria for each training stage are summarised in Table 1. Mice were 2–4 months old when tested (C57 *n* = 16, DBA *n* = 16, CD1 *n* = 15). Data are presented as mean ± standard error of the mean (SEM). *ns* denotes not significant

There were no strain differences between C57, DBA or CD1 mice in the number of sessions taken to reach the criterion on rCPT training stage 1 or 2 (see Fig. 2): stage 1 (*χ*^2^(2) = 0.53, *p* = 0.77, min 2, max 10), stage 2 (*χ*^2^(2) = 3.10, *p* = 0.21, min 1, max 4).

#### rCPT training stage 3: discrimination of S+ and single S**−**

No CD1 animals were able to reach the stage 3 criterion within 19 sessions using baseline parameter settings of a 2-s SD and a 5-s ITI. Moreover, a one-tailed one-sample *t* test showed average *d*′ of the last three sessions in the baseline setting not to be significantly higher than the chance level of 0 (*t*(14) = 2.05, *p* = 0.118, mean *d*′ 0.09, SD 0.17). After a further 13 training sessions using parameter settings with lower attentional load (3-s SD and 4-s ITI), CD1 mice were eventually shown to discriminate S+ and S**−** stimuli slightly but significantly better than chance levels (*t*(14) = 4.45, *p* = 0.002, mean *d*′ 0.15, SD 0.13 across the last three sessions). However, even under easier parameter settings, no CD1 mice ever reached the stage 3 performance criterion (*d*′ ≥ 0.6). Testing of CD1 on the rCPT was thus discontinued and the study focussed on C57 and DBA strains.

**Fig. 3:**
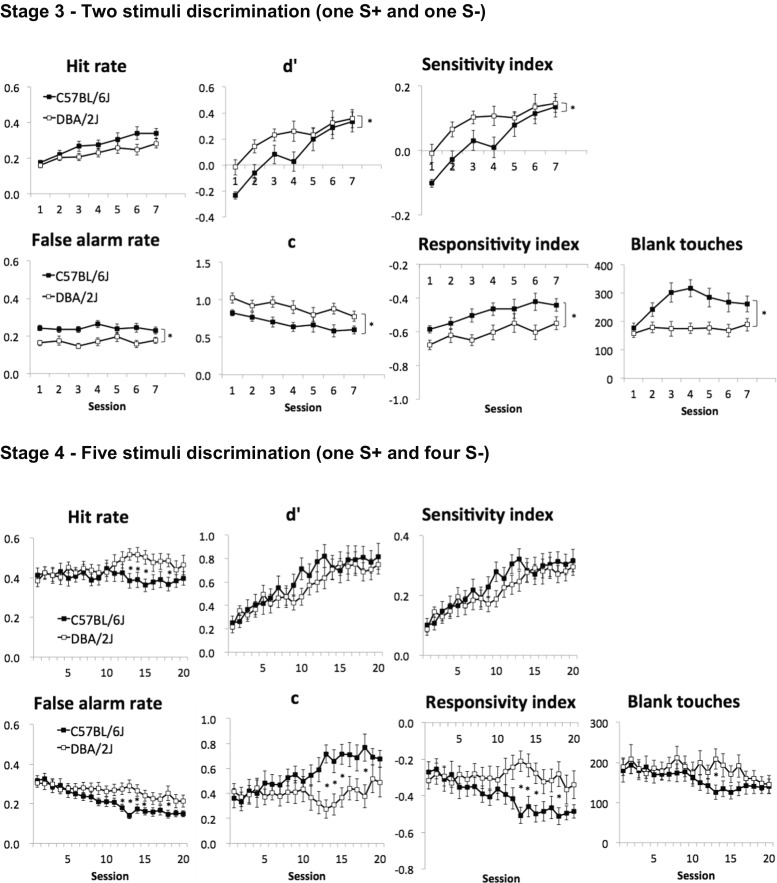
Acquisition of rCPT in stages 3 and 4. All mice were trained at stages 3 and 4 for a minimum of 7 and 20 sessions, respectively. Only these minimal-session acquisition data are shown here in which all animals were being tested at a particular stage (e.g., before some animals reached the criterion). Data are presented as mean ± standard error of the mean (SEM). * denotes *p* < 0.05 in the main effect or simple main effects of strain

In contrast, both C57 and DBA mice were able to acquire stage 3 without a significant strain difference in the number of sessions taken to reach criterion (see Fig. 2; *U* = 121.0, *p* = 0.79, min 6, max 33). Over the course of stage 3 training, HR increased in both C57 and DBA mice across sessions, while FAR did not change (main effect of session: *F*(3.9, 116.3) = 17.27, *p* < 0.001; *F*(6, 180) < 1, ns, respectively). The sensitivity across all training sessions at stage 3 was significantly lower in C57 relative to DBA mice (main effect of strain in *d*′ and SI: *F*(1, 30) = 26.29, *p* < 0.001; *F*(1, 30) = 27.27, *p* < 0.001, respectively). However, this strain difference in sensitivity appeared related to an initial bias of C57 animals towards the novel S**−** and not to the acquisition rate or asymptotic performance. Indeed, on the first session of stage 3, sensitivity measures of C57 mice were significantly lower than those of DBA (*d*′: *t*(30) = −3.29, *p* = 0.003; SI: *t*(30) = −2.99, *p* = 0.006; see Fig. 3). This reduced sensitivity was due to a significantly higher FAR (e.g. preference for S**−**) in C57 relative to DBA mice, with no difference in HR (*t*(30) = 3.92, *p* < 0.001 and *t*(30) = 0.79, *p* = 0.437, respectively). Sensitivity measures gradually increased in parallel across the two strains (*d*′ and SI, main effect of session: *F*(6, 180) = 14.79, *p* < 0.001; *F*(6, 180) = 14.54, *p* < 0.001, respectively), and the average performance of the last three sessions on stage 3 was not different between DBA and C57 mice (*d*′: *t*(30) = −0.37, *p* = 0.716, SI: *t*(30) = −0.50, *p* = 0.621).

Response bias measures showed that a more liberal response criterion emerged across training sessions in both C57 and DBA mice (*c* and RI, main effect of session: *F*(6, 180) = 6.08, *p* < 0.001; *F*(3.9, 117.9) = 7.00, *p* < 0.001, respectively). C57 had higher FAR (main effect of strain: *F*(1, 30) = 12.54, *p* = 0.001) and tended to have a higher response rate than DBA (main effect of strain in *c* and RI: *F*(1, 30) = 6.93, *p* = 0.013; *F*(1, 30) = 6.48, *p* = 0.016, respectively). C57 emitted more blank screen touches than DBA (main effect of strain: *F*(1, 30) = 12.65, *p* = 0.001), but neither an interaction nor a main effect of session was found (both *p* < 0.05). There were no other significant main effects or strain × session interactions.

#### rCPT training stage 4: discrimination of S+ and four novel S**−** leading to asymptotic ‘baseline’ rCPT performance

There was no significant difference between C57 and DBA in the number of sessions taken to reach the stage 4 criterion (see Fig. 2; *U* = 94.5, *p* = 0.45, min 4, max 34). As animals were trained on stage 4, HR did not change in either strain (multivariate simple main effects of session: C57, *p* = 0.962; DBA, *p* = 0.605). This was not unexpected as animals had been trained using the same S+ in stage 3. On the other hand, the initially high FAR (responses to four novel S**−** stimuli) decreased across sessions in both strains (multivariate simple main effects of session: C57, *p* = 0.043; DBA, *p* = 0.001). The stably high HR relative to decreasing FAR led to significant increases in the sensitivity measures across sessions in both C57 and DBA mice (main effect of session in *d*′ and SI: *F*(8.8, 264.6) = 15.70, *p* < 0.001; *F*(9.25, 277.5) = 16.62, *p* < 0.001, respectively) indicating that animals were improving in task performance. There were no differences in the sensitivity measures between C57 and DBA strains (main effects of strain and strain by session interactions: all *p* > 0.1).

Significant strain × session interactions were found for HR and FAR (see Fig. 3; *F*(7.6, 226.6) = 2.51, *p* = 0.014; *F*(7.7, 232.4) = 3.17, *p* = 0.002, respectively). Subsequent simple effects analysis revealed that both HR and FAR were both higher in DBA relative to C57 animals in later training sessions (at sessions 13–15 and 18 in HR; at 12–15 and 19 in FAR; all *p* < 0.05). This increased level of overall responding in DBA mice as stage 4 training progressed was also reflected in response bias measures and blank touches during the ITI (see Fig. 3 for *c*, RI and blank touches; strain × session interactions, simple main effects of strain at sessions 13–15 and 18 in *c* and RI, at session 13 in blank touches, all *p* < 0.05). No other main effects or interactions were significant in stage 4 (all *p* > 0.1).

#### Baseline rCPT performance and within-session time bin analysis

**Fig. 4:**
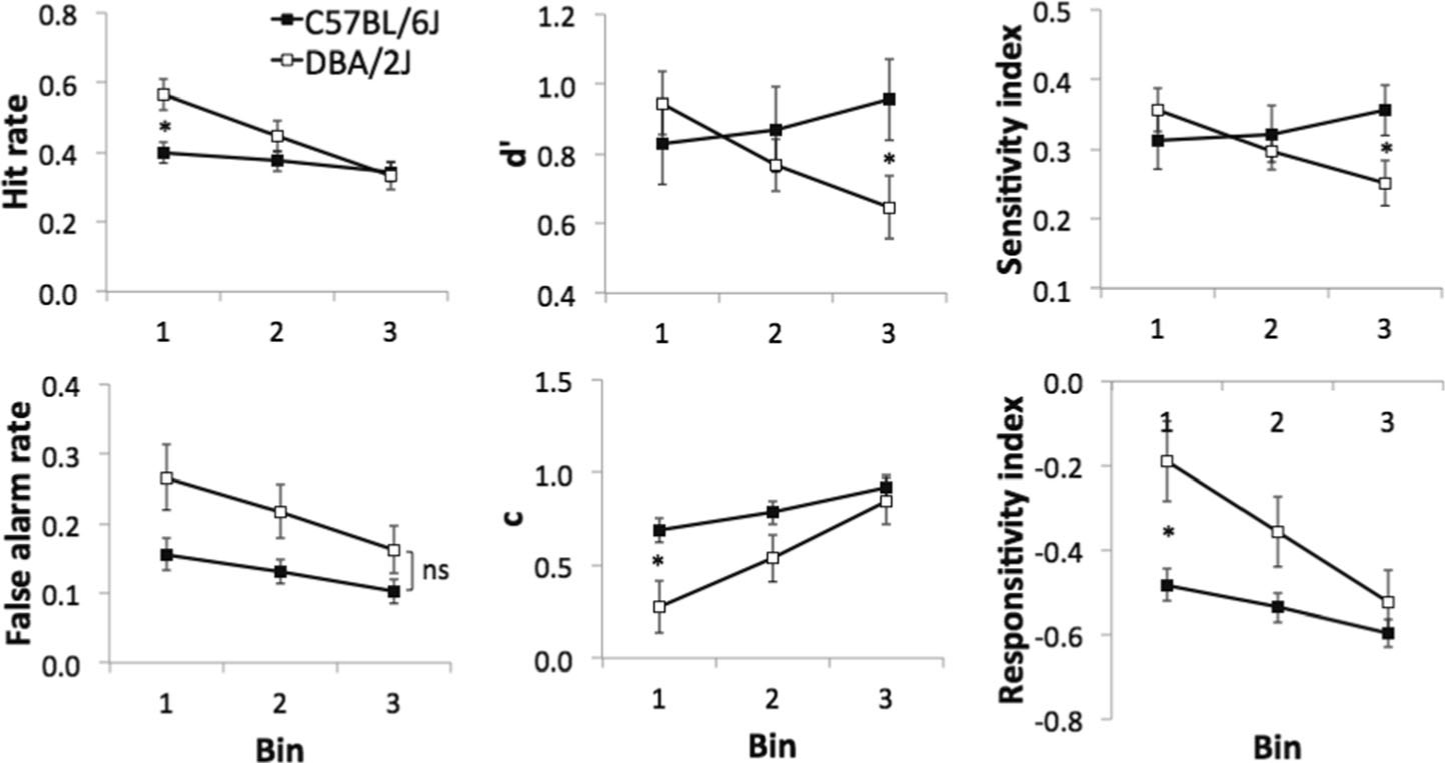
Time bin analysis in rCPT stage 4. Each 45-min session was divided into three equal time bins of 15 min. The average of the final five sessions at stage 4 was used for analysis. There were no differences between strains in any measures without dividing into bins (all *p* > 0.05). Data are presented as mean ± standard error of the mean (SEM). * denotes *p* < 0.05 in the simple main effects of strain

The average of the last five sessions of rCPT stage 4 training was used to assess asymptotic performance levels of C57 and DBA mice (see Fig. 3). There were no significant differences between C57 and DBA strains in HR, FAR, sensitivity, response bias or ‘blank’ ITI touch measures (all *p* > 0.05). To assess sustained attention, performance changes across the session were analysed by dividing sessions into three equal time bins of 15 min on the last five sessions of stage 4 (see Fig. 4). When sessions were divided into time bins, there was a significant bin × strain interaction (*F*(2, 58) = 15.99, *p* < 0.001). For HR, DBA was significantly higher than C57 only at the first bin (*F*(1, 29) = 9.67, *p* = 0.004) and decreased over time bins (*F*(2, 28) = 38.28, *p* < 0.001), while that of C57 did not change (*F*(2, 28) = 2.49, *p* = 0.101).

With respect to FAR, there was a significant main effect of bin (*F*(1.6, 46.0) = 24.85, *p* < 0.001), but no interaction or main effect of strain was found (all *p* > 0.05). These changes in HR and FAR were reflected in sensitivity and bias measures. Similar to HR, there were bin × strain interactions for both sensitivity measures (*d*′ and SI: *F*(1.6, 47.5) = 10.73, *p* < 0.001; *F*(1.57, 45.6) = 11.78, *p* < 0.001, respectively). Simple main effects analysis showed that the sensitivity of DBA decreased over bins (*d*′: *F*(2, 28) = 8.93, *p* = 0.001, SI: *F*(2, 28) = 10.75, *p* < 0.001), but that of C57 did not change (*d*′: *F*(2, 28) = 2.51, *p* = 0.099, SI: *F*(2, 28) = 3.32, *p* = 0.051). The sensitivity of DBA was significantly lower than C57 only in the third bin (*d*′: *F*(1, 29) = 4.33, *p* = 0.046, SI: *F*(1, 29) = 4.57, *p* = 0.041). There were also significant bin × strain interactions for response bias (*c* and RI: *F*(1.16, 46.9) = 9.05, *p* = 0.001; *F*(1.57, 45.6) = 10.05, *p* = 0.001, respectively) and simple main effects analysis revealed that DBA mice had a significantly lower response criterion than C57 mice, only during the first bin (*c* and RI: *F*(1, 29) = 7.61, *p* = 0.010; *F*(1, 29) = 8.51, *p* = 0.007, respectively). Both strains became more conservative across time bins (multivariate simple main effects of bin: all *p* < 0.05).

Response and reward collection latencies were compared between strains using the average of the last five sessions in stage 4. All latencies were longer in DBA relative to C57: correct response latency (*t*(29) = −2.47, *p* = 0.020; C57: *M* 1.11, SD 0.1; DBA: *M* 1.22, SD 0.1), incorrect response latency (*t*(29) = −2.46, *p* = 0.020; C57: *M* 1.06, SD 0.1; DBA: *M* 1.16, SD 0.1) and reward collection latency (*U* = 18.0, *p* < 0.001; C57: *M* 1.80, SD 0.4; DBA: *M* 2.73, SD 1.0).

#### Summary of rCPT training stages and baseline performance

Overall, it took a median of 28 sessions (min 18, max 59, *M* 32, SD 11.2) for C57 and DBA mice to complete all four training stages. More specifically, there were no differences between C57, DBA and CD1 mice in acquisition of rCPT training stage 1 or 2 (simple detection of white square or S+). However, CD1 mice failed to attain the acquisition criterion of rCPT training stage 3 (discrimination of single S+ and S**−**), performing only slightly above chance under reduced attentional load. Both C57 and DBA mice showed successful acquisition of rCPT over training stages 3 and 4, with significant increases in sensitivity measures (*d*′ and SI) across sessions. This was mainly driven by increasing HR (learning of S+) in stage 3, but by decreasing FAR (inhibiting responses to four novel S**−**) in stage 4. While response biases were different, and even opposing, between strains during the acquisition of stages 3 and 4, sensitivity measures were not, suggestive of the independence of SDT-derived sensitivity and response bias measures.

Despite certain strain differences observed during acquisition of rCPT in stages 3 and 4, no asymptotic performance differences in ‘baseline’ rCPT parameters were observed. Finally, when within-session performance was analysed, DBA mice showed reductions in sensitivity over time (C57 mice did not) suggesting differences in sustained attentional function between strains.

### rCPT probe tests

Following acquisition of the rCPT, we investigated how varying task parameters might differentially alter the performance of the mouse strains. It should be noted that, from the start of the probe test sessions, 4 C57 out of 16, and all 13 DBA mice, were single-housed due to intra-cage fighting. One DBA mouse died between probes 1 and 2. Moreover, non-parametric SDT measurements, SI and RI, were not different from parametric measurements, *d*′ and *c*, in terms of gross visual inspection and statistical significances in stages 3 and 4. This pattern was repeated in the probe test sessions —thus, for economy of presentation, only *d*′ and *c* indices are presented.

**Fig. 5:**
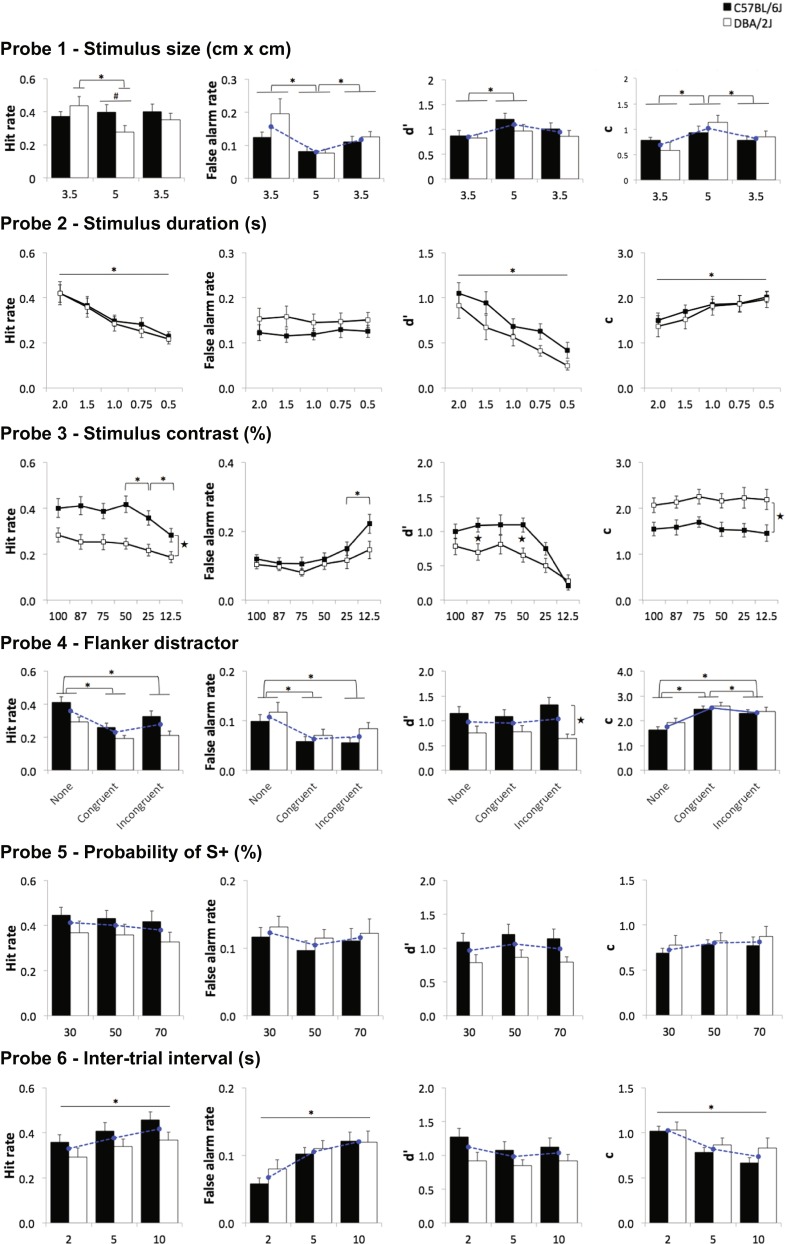
rCPT probe tests. Mice were 5–8 months old when tested (C57 *n* = 16, DBA *n* = 12–13). Four C57 and all DBA were singly housed. *Dotted lines* (in *blue*) represent the average of two strains when there was no difference between two strains, to show the main effect of changes by manipulations. * denotes *p* < 0.05 in the main or simple effects of probe condition, ★ denotes *p* < 0.05 in the main or simple effects of strain, # denotes *p* < 0.1 in simple effects of strain in probe 1

In probe 1 (stimulus size, see Fig. 5 first row), the baseline stimulus size of 3.5 × 3.5 cm was increased to 5 × 5 cm and then returned back to 3.5 × 3.5 cm for the S+ and all S**−** stimuli. There was a significant size × strain interaction in HR (*F*(1.6, 42.7) = 4.66, *p* = 0.021). Simple main effects analysis showed that DBA mice had a significantly lower HR with the larger-sized stimuli relative to the smaller baseline stimuli (*p* = 0.015); the HR of C57 mice did not change across sizes (*F*(2, 26) < 1, ns). There was also a trend that the HR of DBA was lower than C57 at the larger stimulus size (*F*(1, 27) = 3.86, *p* = 0.060), but not at the baseline size (*p* > 0.2). FAR was lower at the large size compared to the baseline size in both strains (main effect of size: *F*(1.2, 33.1) = 8.42, *p* = 0.001; pairwise comparisons: *p* < 0.01), but there was neither a main effect of strain nor a size × strain interaction. Both *d*′ and *c* were significantly higher with the large stimulus size (main effect of size: *p* < 0.01; pairwise comparisons: *p* < 0.05), but these effects were not different between strains (main effect of strain and strain by size interaction: all *p* > 0.1). Thus, larger stimulus size resulted in an increase in sensitivity across both C57 and DBA strains, but also reduced their overall propensity to respond.

In probe 2 (SD, see Fig. 5 second row), HR and *d*′ decreased with reducing SD (*F*(2.0, 52.2) = 56.24, *p* < 0.001; *F*(4, 104) = 29.42, *p* < 0.001, respectively), while FAR did not change (*F*(4, 104) < 1, ns) in both strains. The mice responded significantly less (higher criterion) for stimuli of shorter SD (main effect of SD in *c*: *F*(4, 104) = 20.21, *p* < 0.001). There were no other main effects or interactions. Thus, reducing SD resulted in a decrease in sensitivity across both C57 and DBA strains due to a reduction in hit rate for the S+.

In probe 3 (stimulus contrast, see Fig. 5 third row), reducing stimulus contrast significantly decreased HR and increased FAR (main effect of contrast: *F*(2.8, 73.4) = 13.86, *p* < 0.001; *F*(2.5, 66.2) = 15.18, *p* < 0.001, respectively), particularly under the lower contrast conditions. Specifically, pairwise comparisons between neighbouring contrasts showed significant differences between 50–25 and 25–12.5 % in HR (both, *p* < 0.005) and between 25 and 12.5 % in FAR (*p* < 0.05), but not between others. DBA showed an overall lower HR relative to C57 (main effect of strain: *F*(1, 26) = 9.80, *p* = 0.004; interaction *p* > 0.1), but was not different from C57 in FAR (main effect of strain: *F*(1, 26) = 1.83, *p* = 0.188); interaction *p* > 0.1). There was a significant strain by contrast interaction in *d*′ (*F*(5, 130) = 2.84, *p* = 0.018). Simple main effects analysis revealed the *d*′ of each strain decreased over contrasts (C57: *F*(5, 22) = 15.14, *p* < 0.001; DBA: *F*(5, 22) = 3.53, *p* = 0.017), and the *d*′ of DBA was significantly lower than that of C57 at two mid-contrast levels (both 87 and 50 %, *p* < 0.05). DBA mice also exhibited an overall more conservative response criterion relative to C57 mice (main effect of strain in *c*: *F*(1, 26) = 8.93, *p* = 0.006), but this did not change across contrast levels (main effect of contrast and interaction: both *F* < 1, ns). In sum, reducing stimulus contrast led to decreased perceptual sensitivity in both strains. DBA animals showed greater decreases in sensitivity and a lower propensity to respond relative to C57 mice.

In probe 4 (flanking distractor, see Fig. 5 fourth row), both congruent and incongruent distractor conditions resulted in lower HR and FAR as compared to no distractor (main effect of condition: both, *p* < 0.001; pairwise comparisons between no distractor and either distractor: all *p* < 0.001). There were no differences between the two distractor conditions (pairwise comparisons between congruent and incongruent distractors: all *p* > 0.05). This led to a higher response criterion (lower overall responding) under distractor conditions in both strains (main effect of condition: *F*(2, 52) = 46.13, *p* < 0.001; pairwise comparisons between no distractor and either distractor: all *p* < 0.001). There was no main effect of strain or interaction in HR, FAR and *c* (all *p* > 0.1). However, while *d*′ was unchanged regardless of distractor condition (main effect of distractor: *F*(2, 52) < 1, ns), DBA mice showed lower *d*′ as compared to C57 animals across the distractor probe (main effect of strain: *F*(1, 26) = 7.74, *p* = 0.010). Thus, the addition of congruent or incongruent flanker distractors resulted in a reduction in the propensity to respond in both C57 and DBA mice. DBA mice exhibited an overall lower sensitivity across the entire distractor sessions.

In probe 5 (S+ probability, see Fig. 5 fifth row), there were no main effects or interactions for HR, FAR, *d*′ or *c* (all *p* > 0.05). Blank touches were significantly decreased with increasing S+ probability (*F*(2, 52) = 10.23, *p* < 0.001), but there was no difference between strains (both main effect of strain and interaction, *p* > 0.1).

In probe 6 (ITI, see Fig. 5 sixth row), with longer ITIs, both HR and FAR increased (*F*(1.37, 35.7) = 15.22, *p* < 0.001; *F*(1.5, 38.9) = 24.96, *p* < 0.001, respectively) resulting in an increased willingness to respond to either stimulus type (e.g. decreased c: *F*(1.26, 33.0) = 19.88, *p* < 0.001). There was a non-significant trend for *d*′ to increase under shorter ITI conditions (main effect of ITI: *F*(2, 52) = 3.06, *p* = 0.055). There was no main effect of strain or strain × ITI interaction for *d*′ (*p* > 0.1). As event rate has been demonstrated to have an impact on sensitivity under certain conditions for human CPT (Parasuraman 1979), we further analysed the probe 6 data dividing the session into three 15-min time bins. Replicating the effects observed at the end of rCPT stage 4 training, DBA showed a highly significant reduction in *d*′ across the session that was not observed in C57 mice (strain × time bin interaction: *F*(2, 44) = 18.28, *p* < 0.001). No significant strain × ITI × time bin interaction was found. In sum, increasing the ITI between stimulus presentations resulted in an increase in the overall propensity to respond to these stimuli. DBA mice showed reduced vigilance across the session compared with C57 mice.

### Pairwise visual detection and discrimination

**Fig. 6:**
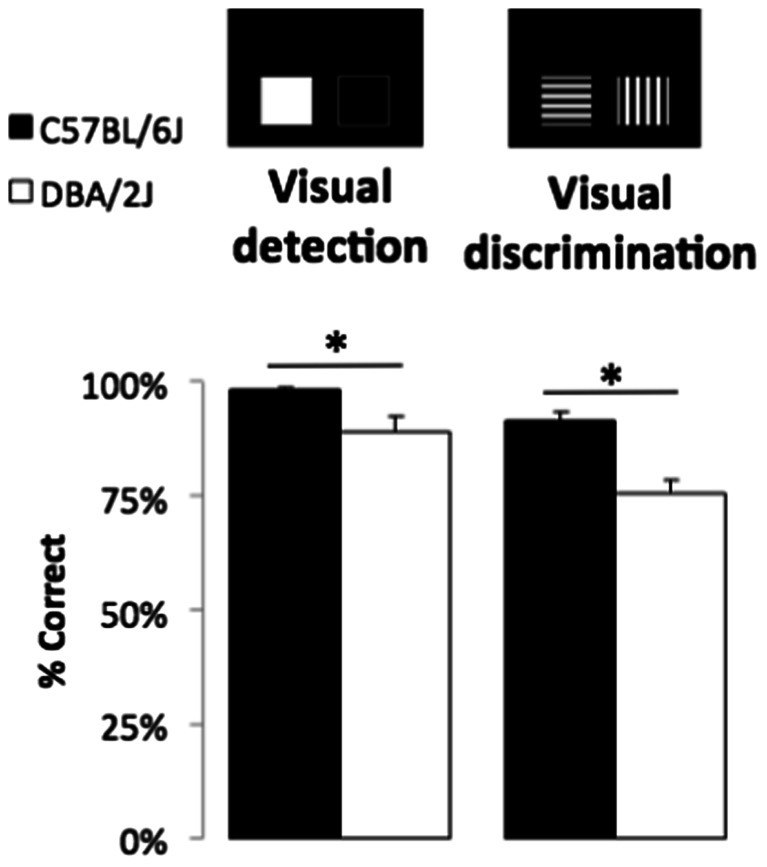
C57BL/6J and DBA2/J on pairwise visual detection and visual discrimination tasks. The pairwise visual detection task required the mice to discriminate between a *white square* and a blank screen. The pairwise visual discrimination task required the mice to discriminate between two luminance-matched visual patterns which were used in the rCPT. C57 and DBA were 9 months old when tested (C57 *n* = 16, DBA *n* = 11). Four C57 and all DBA mice were singly housed

Following rCPT training and probe testing, C57 and DBA mice were assessed in the two-alternative forced choice procedures for visual detection and discrimination at 9 months of age. To minimise the effects of new learning, the same stimuli from stage 4 rCPT were used. The last three sessions of each task procedure were averaged to compare the two strains. The performance of DBA was significantly lower than that of C57 in both visual detection and visual discrimination tasks (see Fig. 6; *U* = 43.0, *p* = 0.023; *t*(25) = 5.10, *p* < 0.001, respectively).

In addition, to gain insight into whether single housing may have affected the task performance of C57, the C57 group was divided into group-housed (*n* = 12) and single-housed groups (*n* = 4) and analysed together with single-housed DBA (*n* = 11). The three groups were significantly different in both visual detection and discrimination tasks (*χ*^2^(2) = 7.13, *p* = 0.028; *χ*^2^(2) = 14.45, *p* = 0.001, respectively): group-housed C57 (median 100 and 96.1 %), single-housed C57 (median 98.3 and 90.5 %) and single-housed DBA (median 93.3 and 75.6 %). Post hoc tests for the Kruskal-Wallis test revealed that the two C57 groups were not different (*p* > 0.1), but the performance of DBA was significantly lower than that of the group-housed C57 (*p* < 0.05). Thus, the observed strain differences on pairwise visual detection and discrimination are likely not driven solely by the effects of single housing.

After failing to reach the stage 3 criteria of the rCPT task, the CD1 group was also tested in the visual discrimination task. There was no significant evidence of learning for nine sessions (main effect of session: *F*(8, 64) < 1, ns; last three session average 55.2 % (SD 5.9 %)). Even after increasing the stimulus size from the original 3.5 × 3.5 to 7 × 7 cm, the performance of CD1 did not improve across 10 sessions (main effect of session: *F*(9, 90) < 1, ns; last three session average 53.2 % (SD 10.5 %)).

### Effects of donepezil on rCPT

Two donepezil i.p. injection schedules were used: the first with saline, 0.03-, 0.1- or 0.3-mg/kg donepezil (schedule 1) and the second, to replicate and extend the initial findings, with saline or 3-mg/kg donepezil (schedule 2). The order of doses was counterbalanced in a Latin square design for each group. Due to atypical behaviours after injection, i.e. immobility, one and three C57 mice were excluded from the analysis in schedules 1 and 2, respectively. In schedule 1, six DBA mice were excluded because they were accidentally given an incorrect dose on one of the sessions. Four C57 and all DBA mice were singly housed during these experiments.

For HR and FAR, there were no consistent three-way interactions across the two dosing schedules 1 and 2. In HR, a strain × dose × SD interaction was significant only in schedule 2 (*F*(1, 21) = 17.55, *p* < 0.001). Decomposition of this interaction revealed that in DBA mice, HR was significantly decreased by 3-mg/kg donepezil relative to vehicle at the 4-s SD (*p* = 0.006) but not at the 1-s SD (*p* > 0.1). In addition, DBA mice had significantly higher HR compared to C57 mice in the saline condition only at the 4-s SD (*p* = 0.006), but not at 1-s SD (*p* > 0.1). No differences were observed in C57 mice (*p* > 0.1). In FAR, a strain × dose × SD interaction was significant only in schedule 1 (*F*(3, 57) = 2.96, *p* = 0.040) with significant simple strain × SD interactions at 0.03-, 0.1- and 0.3-mg/kg doses (all *p* < 0.05). These interactions were explained by significantly higher FAR in DBA relative to C57 animals at 4-s SD (all *p* < 0.05), but no differences at 1-s SD (*p* > 0.1).

**Fig. 7:**
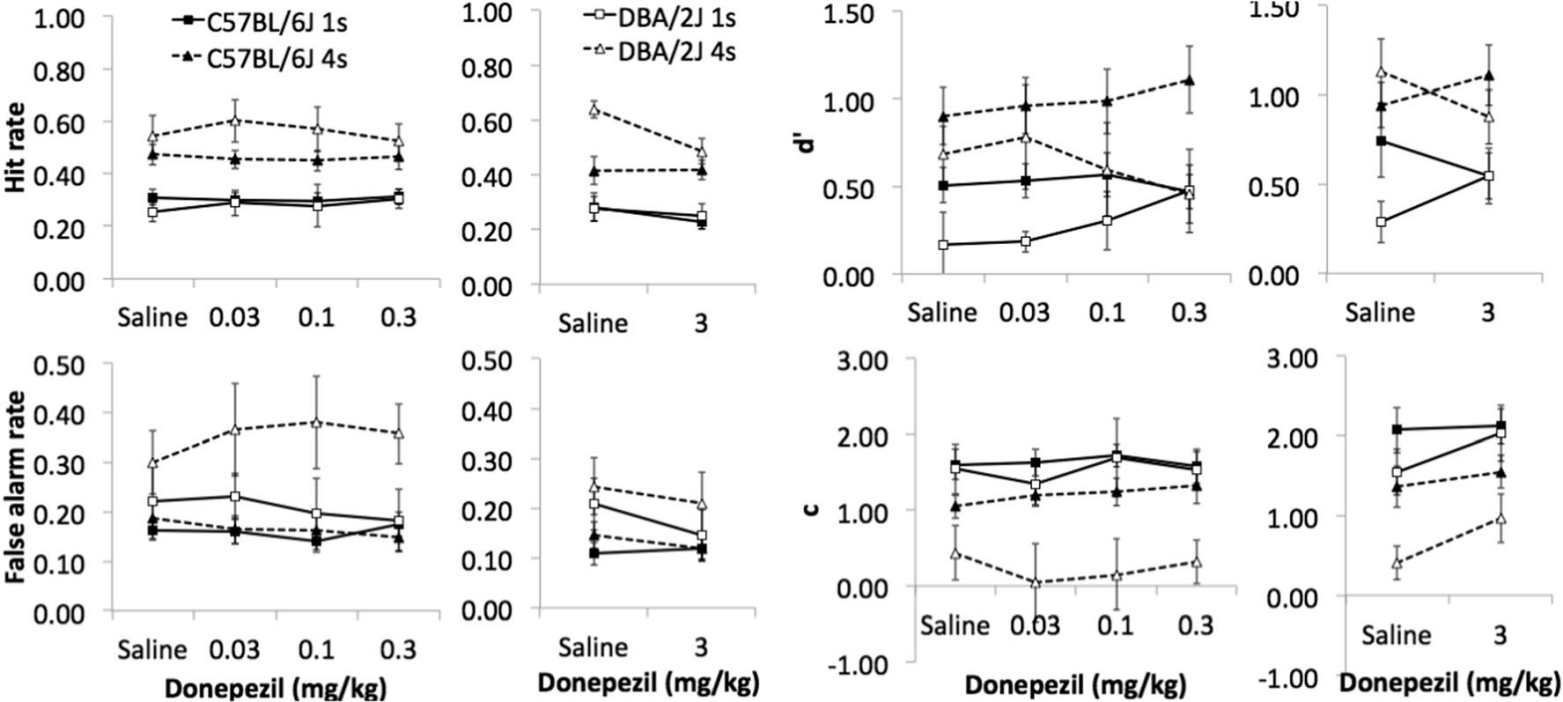
Effects of donepezil on C57BL/6J and DBA2/J. Both strains were 9 months old when tested

Despite the relatively inconsistent effects of donepezil on HR and FAR per se, there were significant strain × dose × SD interactions for sensitivity across both schedules 1 and 2 (see Fig. 7: *F*(3, 57) = 3.70, *p* = 0.017; *F*(1, 21) = 7.20, *p* = 0.014, respectively). In C57 mice, there was no significant dose × SD interaction in either schedule (schedule 1: *F*(3, 42) = 1.03, *p* = 0.387; schedule 2: *F*(1, 12) = 2.05, *p* = 0.178). However, in DBA mice, significant dose × SD interactions were found for both schedules (schedule 1: *F*(3, 15) = 3.92, *p* = 0.0.30; schedule 2: *F*(1, 9) = 9.24, *p* = 0.014). Under vehicle in schedule 2, there was a significant interaction (*F*(1, 21) = 6.99, *p* = 0.015) where second-order simple effects showed only *d*′ of DBA mice to be significantly higher at the longer 4-s SD relative to 1-s SD (*F*(1, 21) = 5.12, *p* < 0.001), while that of C57 was unchanged (*F*(1, 21) = 1.48, *p* = 0.238). Second-order simple effects analysis in DBA in schedule 1 further revealed that the performance of 4-s SD was significantly higher than that of 1-s SD in the saline condition (*p* = 0.036), but not at other doses (all *p* > 0.05). The same analysis of DBA in schedule 2 found that the performance of 4-s SD was higher than that of 1-s SD in both saline and 3-mg/kg donepezil conditions (*p* < 0.001 and *p* = 0.037, respectively). At the highest 0.3 mg/kg in schedule 1, a significant interaction was found (*F*(1, 19) = 8.52, *p* = 0.009) that was driven by a significant elevation of *d*′ at the longer 4-s SD relative to 1-s SD in C57 mice (*F*(1, 19) = 27.89, *p* < 0.001) with no such differences in *d*′ in DBA animals (*F*(1, 19) < 1, ns). No other significant simple interactions were found in other conditions.

For the response bias measure, *c*, there were only significant strain × SD interactions in both schedules 1 and 2 (*F*(1, 19) = 18.83, *p* < 0.001; *F*(1, 21) = 7.65, *p* = 0.012, respectively). Simple effects analysis showed that DBA mice had a lower response criterion (higher overall response rate) than C57 mice at 4-s SD (schedule 1: *F*(1, 19) = 8.60, *p* = 0.009; schedule 2: *F*(1, 21) = 6.45, *p* = 0.019), but not at 1-s SD (both, *p* > 0.1). These effects were independent of changes in donepezil dose.

In summary, donepezil exerted a consistently greater effect on DBA relative to C57 mice across both dosing schedules. DBA mice showed significantly reduced sensitivity for shorter 1-s SD stimuli compared to 4-s SD stimuli following vehicle injections. This difference in sensitivity was reduced by donepezil treatment. These SD-specific effects of donepezil in DBA mice are driven both by the tendency towards a dose-dependent increase in sensitivity for more difficult 1-s SD stimuli, as well as a dose-dependent decrease in sensitivity for easier 4-s SD stimuli. The opposite tendency was observed in C57 mice with higher doses of donepezil increasing the differences between 4- and 1-s SD performance (e.g. tending to enhance 4-s SD performance while tending to reduce 1-s SD performance).

## Discussion

The present study has shown that a new touchscreen-based test of attentional function, the rodent continuous performance test (rCPT), can be used effectively to define specific phenotypes in mouse strains and to provide a sensitive assay following treatment with an anti-cholinesterase. The task is similar to the human CPT in that it requires the detection, discrimination and appropriate response (touch) to a brief visual target stimulus, as well as inhibition of responses to multiple non-target stimuli, presented sequentially over a number of trials.

Manipulation of rCPT parameters produced effects in mice similar to those expected in humans, providing behavioural validation of the task. Increased stimulus size (probe 1), which enhances salience and resolvability, led to improved perceptual sensitivity. This enhancement of *d*′ was due mostly to a differential decrease in the FAR rather than increases in HR. At first glance, it may seem unexpected that the HR is unchanged (C57) or even decreased (DBA) when mice are confronted with a more salient and resolvable stimulus. However, it should be noted that increased overall brightness and spatial frequency alter the visual properties of the stimulus away from the typical S+ target to which the animals have been extensive trained/tuned and, thus, will likely be perceived as slightly different. Conversely, reducing stimulus duration (probe 2) or contrast (probe 3) caused the expected decreases in sensitivity related to increases in attentional and perceptual load. Changes in target S+ stimulus probability (probe 5) or stimulus presentation rate (probe 6) led to a reduction in blank (ITI) touches and longer ITI increased the propensity to respond, in the absence of effects on *d*′. Indeed, a common effect of manipulating the probability and frequency of target appearance (and thus also likely, reward density) is to alter response bias, without affecting overall perceptual sensitivity (Macmillan and Creelman 2004). While S+ probability and stimulus event rate have been demonstrated to affect *d*′ under particular conditions in human CPTs (Parasuraman 1979), it is possible that the ‘baseline’ rCPT conditions in the present study were difficult enough for mice that the changes in parameters were not extreme enough to induce further differences. The use of more extreme values of S+ probability (e.g. 10 %) and/or event rate (e.g. 1/s or 1/30 s) may serve to bring out further effects in perceptual sensitivity. Finally, although a congruency effect (enhanced performance when distractors are congruent versus when they are incongruent) was not observed in the flanking distractor condition (probe 4), the decreased HR and FAR with corresponding increase in response criterion on trials when flanking stimuli were presented indicate that these distractor foils were sufficient to interfere with normal task performance. It should be noted that, distinct from the human task, animals may not necessarily be centrally positioned on every trial, and thus, the impact of flanking stimuli might be expected to be more distracting (e.g. attend or approach the flanking stimulus location). Overall, the results of the set of probe test are generally highly consistent with how such parametric changes impact upon human CPT performance and suggest that the rCPT might be a useful translation test for the assessment of selective attention, as well as for visual perception and response inhibition (Nuechterlein et al. 1983; Mass et al. 2000; Conners et al. 2003; Berwid et al. 2005; Demeter et al. 2008).

### rCPT in C57 and DBA mice: task acquisition

There were no differences between C57 and DBA animals in either the number of sessions to reach criterion or in asymptotic performance/sensitivity levels at any of the four training stages. C57 mice did have significantly lower *d*′ and SI scores relative to DBA animals during the acquisition of rCPT training stage 3. However, this effect was largely due to an initial bias towards choosing the newly introduced S**−** stimulus in C57 mice, despite having similar learning trajectories as DBA mice. It is unclear how such biases emerged, but may relate to strain differences in novelty preference, recognition memory or perceptual bias towards the particular novel S**−** stimulus used (‘snowflake’) exclusively in stage 3. Systematic differences in stimulus preferences between C57 and DBA mice have been reported previously within the touchscreen apparatus (Dickson et al. 2014). Counterbalancing the novel S**−** with S+ (e.g. as done in stage 4) in future experiments may help discern these possibilities.

Despite the absence of asymptotic differences in sensitivity measures, response bias indices varied considerably between C57 and DBA mice across rCPT training stages. During stage 3, C57 animals had a higher overall response rate relative to DBA animals, whereas the opposite pattern was observed during stage 4, particularly in the later sessions of training. A similar effect was observed for the number of blank square touches during the ITI (C57 > DBA at stage 3, DBA > C57 later in stage 4). A possible reason for the observed strain differences in responsivity across stages 3 and 4 may relate to differential changes in motivation. It has previously been shown that, relative to C57BL/6, DBA mice have a higher metabolic rate (Ferguson et al. 2008) and work for and consume significantly more food in a manner not attributable to differences in body weight (Atalayer and Rowland 2010). This suggests that, under similar levels of food restriction, DBA mice may have experienced progressively increased motivation relative to C57 mice across the training stages. This may also help explain the higher intra-cage aggression observed in DBA mice as the experiment progressed beyond stage 4. Alternatively, these results may indicate learning strategy differences between strains as other indices commonly reflecting motivation or speed/accuracy trade-off were not trending in the same direction, e.g. response and reward collection latencies were longer in DBA mice despite having a higher overall response rate relative to C57 animals. Regardless of causes, these data show that the rCPT is sensitive to individual differences in response bias and that such alterations appeared independent of changes in perceptual sensitivity, as predicted by detection theory (Macmillan and Creelman 2004).

### rCPT in C57 and DBA mice: attentional performance

Analysis of performance across the rCPT session revealed key differences between C57 and DBA mice (see Fig. 4). Specifically, the hit rate and sensitivity measures of DBA mice decreased linearly across the session, while those of C57 mice did not. Moreover, although the bias towards responding decreased in both strains, the extent of the decline was greater in DBA animals, due to their differential reduction in hit rate. The effect appeared extremely robust and strain-specific as it was also observed during the final probe test (rCPT probe 6—event rate) conducted several months later and despite differences in the animal housing conditions (e.g. DBA multiply versus singly housed). These strain differences on baseline rCPT performance across the session appear generally consistent with the results from studies using simple detection-based paradigms, but are the first to indicate a specific strain × session time interaction. Greco et al. (2005) showed that C57BL/6N mice were more accurate than DBA/2N mice selectively on probe tests using shorter SDs, thought to increase attentional load in the 5-CSRTT. This finding was replicated in C57BL/6J and DBA/2J mice (Patel et al. 2006). Using C57BL/6J and DBA/2J strains in the 5C-CPT, Young et al. (2009) reported a decrease in sensitivity (SI) over trials across both strains; however, sensitivity was lower in DBA relative to C57 mice when analysed across the entire session. Beyond strain differences, reductions in hit rate and sensitivity are typically observed in human versions of CPT under similar conditions (e.g. sequential presentation of stimuli at a relatively high rate) to those used here in the rCPT (Parasuraman 1979). Moreover, within-session attentional decrements are typically observed in human patients using sustained attentional tasks including CPT (Brazzelli et al. 1994; See et al. 1995; Baddeley et al. 1999; Mass et al. 2000; Berardi et al. 2005). These findings suggest a high translational value of the rCPT for the study of individual differences in sustained attention.

### rCPT in C57 and DBA mice: probe tests

As described above, the results of the rCPT probe tests are largely consistent with those expected based on human CPT studies. Interestingly, across the six probe tests, only three revealed differences between C57 and DBA mice: stimulus size (probe 1), stimulus contrast (probe 3) and flanker distractors (probe 4). Relative to C57 mice, DBA showed a reduced hit rate to larger stimuli; decreases in hit rate, sensitivity (*d*′) and overall response rate (e.g. increased *c*) when faced with reductions in stimulus contrast; and reduced overall *d*′ on sessions that included flanker distractors. These results should perhaps be interpreted with some caution as, at the time of probe testing, all DBAs and four C57 mice had been single-housed. However, the selective effects on probes in which visual properties of stimuli were altered or rendered more difficult to discriminate are highly suggestive of differences in visual capacity between C57 and DBA mice. Indeed, the largest strain difference seen on the rCPT contrast probe is consistent with, and may provide a sensitive behavioural correlate for, observed differences in retinal function and contrast gain control between C57 and DBA mice (Porciatti et al. 2010).

DBA/2J mice are a standard and well-characterized preclinical model of age-related pigmentary glaucoma (McKinnon et al. 2009). It is thus perhaps surprising that deficits in overall sensitivity on rCPT were not observed beyond probes that change the visual properties of the stimuli. On the one hand, our finding that asymptotic %correct of DBA mice was lower than C57 on both simple and luminance-matched pairwise detection and discrimination tasks at about 10 months of age appears consistent with possible visual impairment. In swim test variants of the pairwise visual discrimination paradigm, it was found that DBA mice outperformed C57 at 6 months of age (with acuity estimates 0.37–0.54 cycles/degree [c/d]; Wong and Brown 2006, 2007), whereas performance of DBA animals declined and was lower than C57 at 12, 18 and 24 months (Wong and Brown 2007). On the other hand, studies assessing the optokinetic reflex tracking response as a correlate of visual acuity have shown that, despite progressive increases in intraocular pressure and reductions in ganglion cell density, DBA mice can resolve spatial frequencies approximately 0.3–0.4 c/d at 6 and even up to 12 months of age (Zhou et al. 2009; Burroughs et al. 2011). Moreover, C57 mice have a reported visual acuity of ~0.4 c/d from P26 onwards (Prusky et al. 2004; Prusky and Douglas 2004), and we estimate the acuity requirements of rCPT stimuli to be ~0.1 c/d (assuming, conservatively, that mice position themselves 4 cm away while attending to the screen). These latter findings thus predict that there may be limited strain differences in discrimination on the rCPT using high-contrast stimuli. This is in line with our current rCPT dataset in which DBA mice show no impairments in overall sensitivity on baseline and certain probe tests (SD, ITI, probability) between 4 and 12 months of age.

While it is difficult to reconcile our strain difference observed in pairwise discrimination tasks with the general lack of overall *d*′ differences in rCPT, our probe tests may provide additional insight. The fact that *d*′ was reduced in DBA animals during sessions including flanker visual distractors suggests a deficit in their ability to selectively attend and/or respond appropriately when additional adjacent stimuli are present. Alternatively, the fact that DBA mice showed decreased hit and overall response rates when presented with larger stimuli, despite larger stimuli having decreased spatial frequency and increased resolvability, may suggest a deficit in perceptual generalization. Such a generalization deficit may also contribute to the observed DBA sensitivity deficits on contrast and distractor probes in which the visual context was altered. Generalization deficits have been observed in DBA relative to C57 mice in other learning contexts (Waddell et al. 2004) and may have attenuated learning the pairwise discrimination procedure following extensive training on rCPT. Finally, optimal performance on pairwise discrimination tasks may require animals to increase their distance from the screen in order to simultaneously ‘sample’ both stimuli. This would reduce stimulus resolvability and potentially accentuate strain differences in visual acuity. Further research is needed to distinguish such alternatives based on attention, generalization or visual capacity. However, information gained from rCPT probes can help reveal the nature of behavioural impairments and generate new testable hypotheses.

### rCPT in CD1 mice: visual impairments

The most obvious strain difference found was that CD1 mice were unable to reach the acquisition criteria of the rCPT defined by *d*′ in stage 3, which requires visual discrimination of successively presented S+ and a novel, luminance-matched S**−** stimulus. This failure to progress occurred despite the fact that CD1 mice performed similarly to C57 and DBA animals in rCPT training stages 1 and 2 (which only requires visual detection). CD1 mice also failed to learn the pairwise visual discrimination task with the same visual stimuli used in rCPT stage 3, as well as with larger more salient ones having lower spatial frequency. A previous study found that CD1 mice can successfully acquire the 5-CSRTT based on brief light stimulus detection within an operant chamber (Oliver et al. 2009), consistent with their relatively unimpaired performance on rCPT training stages 1 and 2. Other studies have also observed that CD1 mice can learn the Morris water maze task under high illumination conditions, a task requiring discrimination of distal extramaze visual cues (Adams et al. 2002). However, Abdeljalil et al. (2005) reported that CD1 mice did not show any optomotor responses (at any spatial frequency) within a rotating drum with black and white stripes. Taken together, these results suggest that the CD1 group’s performance deficit is likely due to their poor perceptual acuity within the touchscreen apparatus.

### Effects of donepezil on rCPT performance

Donepezil generally exerted greater effects in DBA relative to C57 mice. Specifically, the major effects involved dose-dependent interactions of donepezil with stimulus duration in DBA animals: increasing doses of donepezil tended to improve performance under more difficult 1-s SD but to decrease performance under less taxing 4-s SD conditions. This result was consistent across the two dosing experiments. To a lesser extent, the opposite pattern was observed for C57 animals (e.g. reduced performance at 1-s SD and improved performance at 4-s SD). Overall, these findings are consistent with, and may add insight to, the existing literature examining cholinergic differences between the two strains. DBA mice have been reported to exhibit a higher number of forebrain cholinergic neurons and increased acetylcholine turnover rates in the hippocampus, caudate and frontal-parietal cortex than C57 mice (Durkin et al. 1977; Bentivoglio et al. 1994). Previous studies have also generally found that DBA mice are behaviourally more sensitive to cholinergic manipulations as compared to C57 animals. For example, administration of the cholinesterase inhibitor, physostigmine, dose-dependently improved retention of an inhibitory avoidance response of DBA mice to a greater extent that in C57 mice (Castellano et al. 1996). Pretreatment with a psychostimulant, cocaine, potentiated the mnemonic effects of physostigmine in C57 mice, while antagonizing them in DBA mice. Further, scopolamine caused greater reductions in performance in DBA versus C57 mice on delayed non-matching-to-position and radial maze tasks (Estapé and Steckler 2002; Ammassari-Teule and Caprioli 1985). In contrast, Romberg et al. (2011) showed that donepezil had no effect on the performance of C57-background mice in the 5-CSRTT, in line with our general lack of donepezil effects for this strain in rCPT.

While the enhanced sensitivity of DBA mice to cholinergic manipulations appears consistent with a variety of studies, the opposing effects of donepezil at different stimulus durations in DBA mice (e.g. trend towards improvements at shorter SD and towards impairments at longer SD) require further explanation. This result might well reflect an inverted U function for the impact of cholinergic activity on attentional function. In other words, increasing doses of donepezil may help to enhance performance for stimuli requiring greater attentional resources but act to disrupt performance relative to vehicle controls for easier stimuli. Specific to the present study, as donepezil appeared to act to help differentially reduce FAR (relative to HR), this might have resulted in an increase in *d*′ when FAR levels are high (e.g. at shorter SDs), but to a paradoxical decrease in *d*′ when FAR is near floor levels (with HR decreasing slightly). This may also help explain the general lack of effects of donepezil on C57 animals who exhibited lower FAR relative to DBA mice. Such dose- and/or task difficulty-dependent inverted U curves have been observed following administration of cholinesterase inhibitors and other cholinergic mimetic compounds in rats, non-human primates and humans performing tasks assessing cognitive functions such as attention and working memory (Aston-Jones et al. 1999; Bentley et al. 2011; Van Dam et al. 2005; Robbins 2000). Finally, it is important to note that the fact that opposing effects at different SDs were observed, it is unlikely that changes are simply related to donepezil-induced alterations in visual capacity per se. Taken together, our results are consistent with the suggestion that tonic acetylcholine levels may regulate optimal rCPT performance through alterations in cognitive/attentional control at varying levels of attentional load.

### Comparison with other rodent tests of attentional function

Other automated tests, e.g. 5-CSRTT, 5C-CPT and SAT/dSAT, are available for assessing attention in the mouse and have yielded a great deal of valuable data (Humby et al. 1999; Young et al. 2009; Romberg et al. 2011; St. Peters et al. 2011; Parikh et al. 2013; van Enkhuizen et al. 2014). These tests require subjects to detect and/or spatially localise a simple light stimulus. By contrast, similar to a number of human CPT variants, successful performance on the rCPT requires both detection and discrimination of a visual pattern from a set of luminance-matched non-target stimuli with overlapping features. Simple detection and/or orientation towards a light may recruit neural pathways and cognitive/perceptual processes distinct from those required for more complex visual discrimination (Lashley 1931; Petruno et al. 2013; Schneider 1969; Pöppel et al. 1973; Stoerig et al. 1985). Thus, the 5-CSRTT, 5C-CPT and SAT for the rodent may tap different circuits to those tapped by rodent and human CPT. In addition, the flexibility of the touchscreen platform allows for a variety of perceptual and/or behavioural challenges, such as flanking congruent and incongruent distractors that are more difficult to achieve using other methodologies.

From a practical point of view, the training period of the rCPT was comparable to and generally considerably shorter than other mouse attentional tasks. In the present study, it took a median of 28 sessions (min 18, max 59, *M* 32, SD 11.2) for C57 and DBA strains to finish all training stages of the rCPT (up to 2-s SD with one S+ and four S**−**), excluding two habituation sessions. This was similar to the training period to 2-s SD of other mouse 5-CSRTT studies (Humby et al. 1999; Romberg et al. 2011), which did not include initial shaping or pretraining to operant chambers in their estimates (typically 5–10 sessions). A recent mouse 5C-CPT paper (van Enkhuizen et al. 2014) described that it took up to 70 sessions for 5C-CPT training. In the first mouse SAT paper (St. Peters et al. 2011), it took an average of approximately 50 sessions to reach the final stage to training. Moreover, it is possible to achieve even shorter training times in the rCPT if mice are trained up to stage 3, with only one S+ and one S**−** stimulus (in the current study: median 19, min 11, max 38, *M* 20, SD 6.6 sessions), which has all components of full rCPT (with the exception of multiple foils), and all probes and behavioural challenges are still possible within this reduced version of the task.

## Conclusions

The rCPT, which was originally developed in rats (Mar et al., unpublished) as an analogue of human CPT, has been successfully adapted for use in mice. Mouse performance on the rCPT was appropriately altered by a number of parametric and behavioural manipulations to help interpret task results. The task was further able to sensitively detect both major and more subtle differences in attentional performance, executive function and visual capacity between DBA, C57 and CD1 mouse strains and following pharmacological treatment with the cholinesterase inhibitor, donepezil. The rCPT may prove to be a valuable tool for assessing rodent disease models and translating the findings to human patients.
